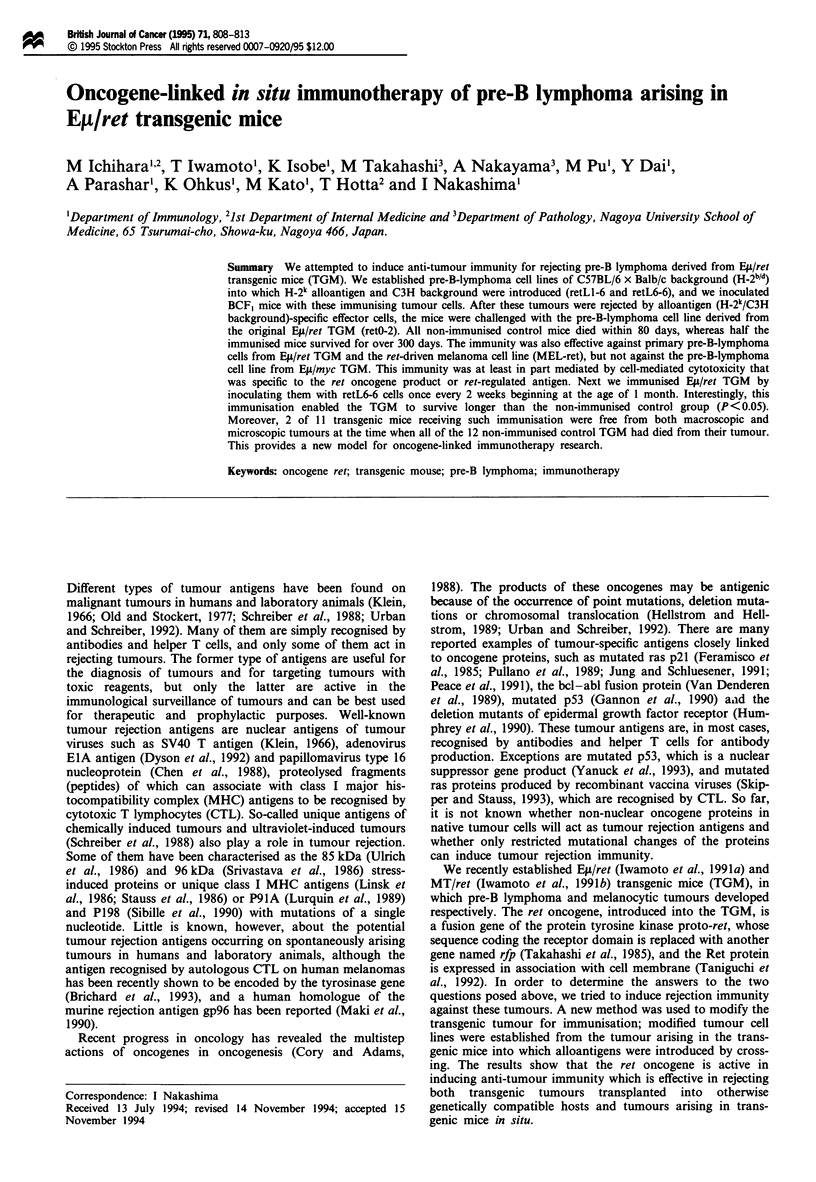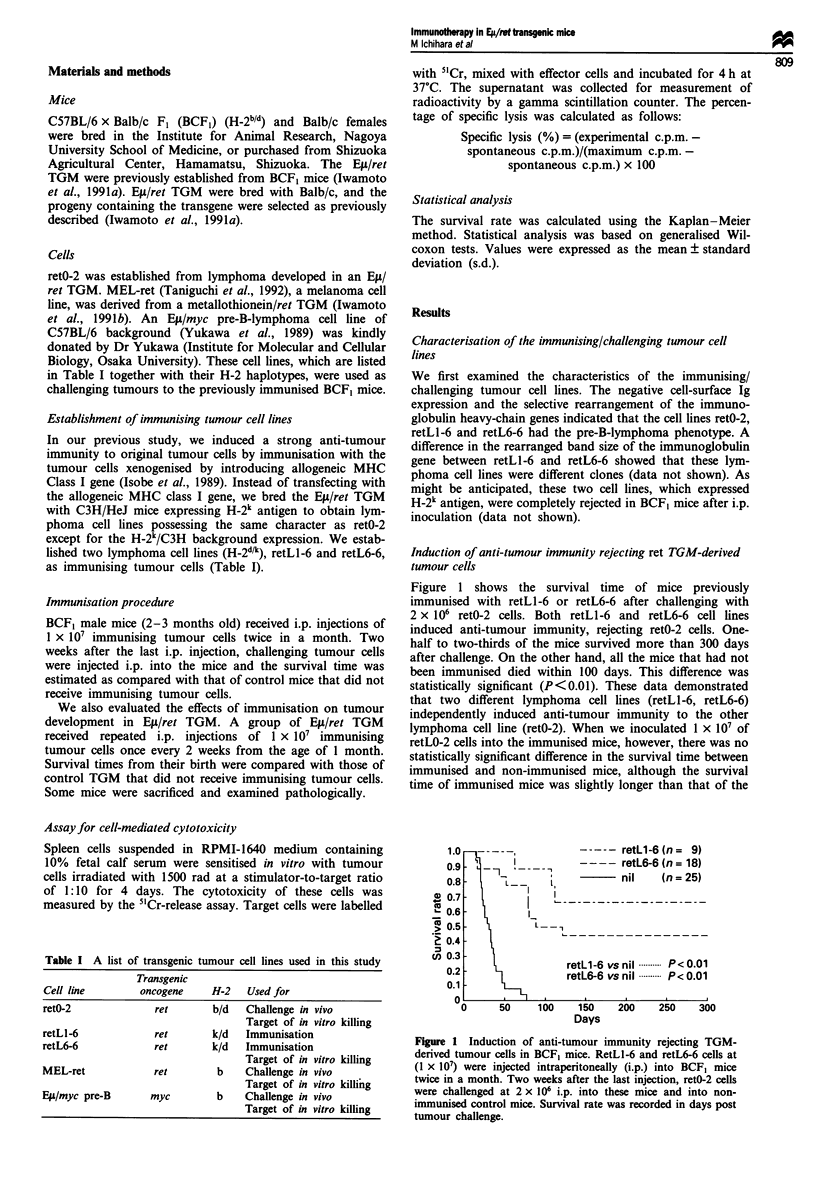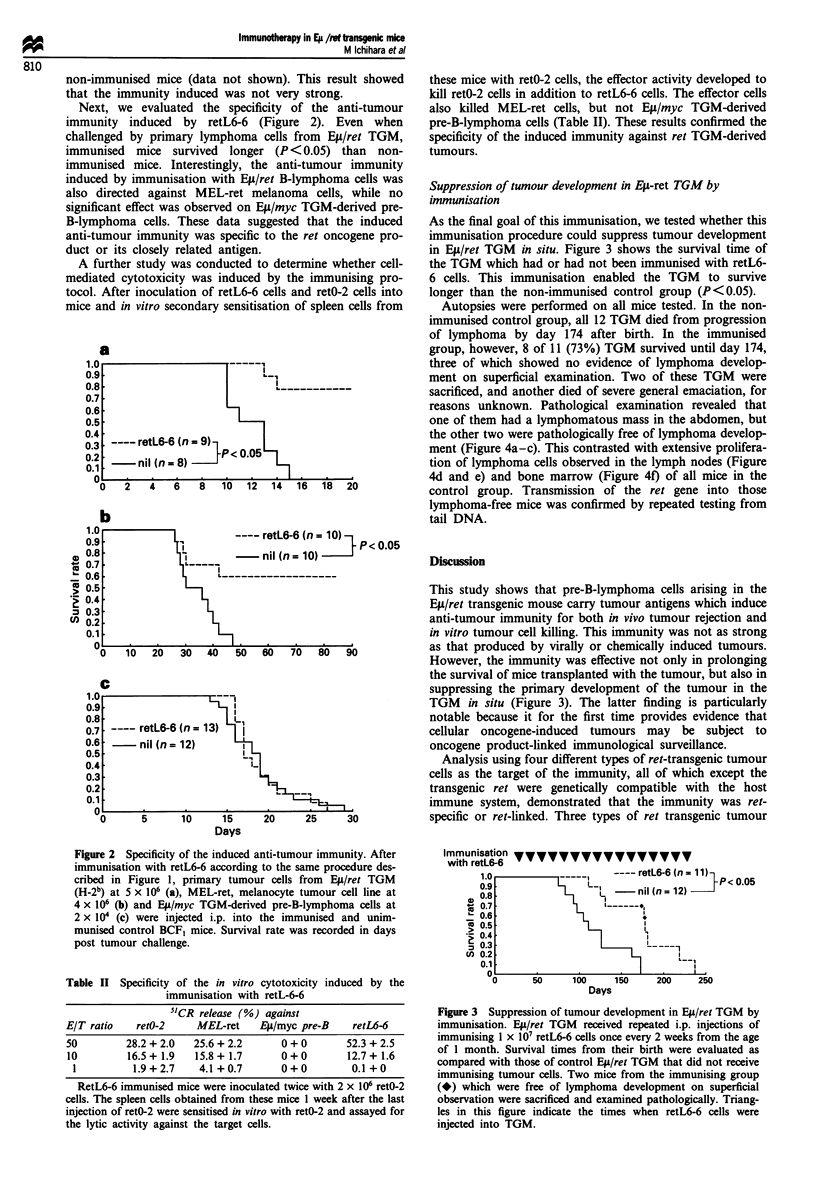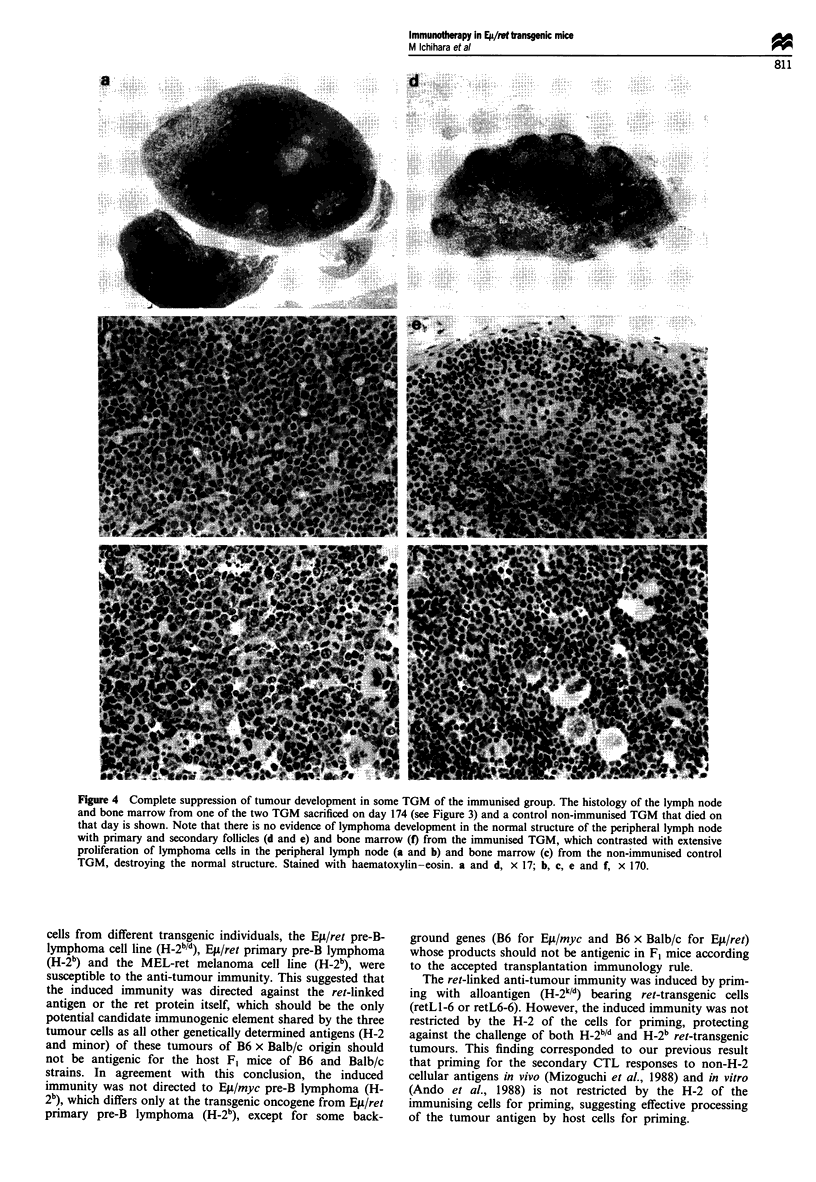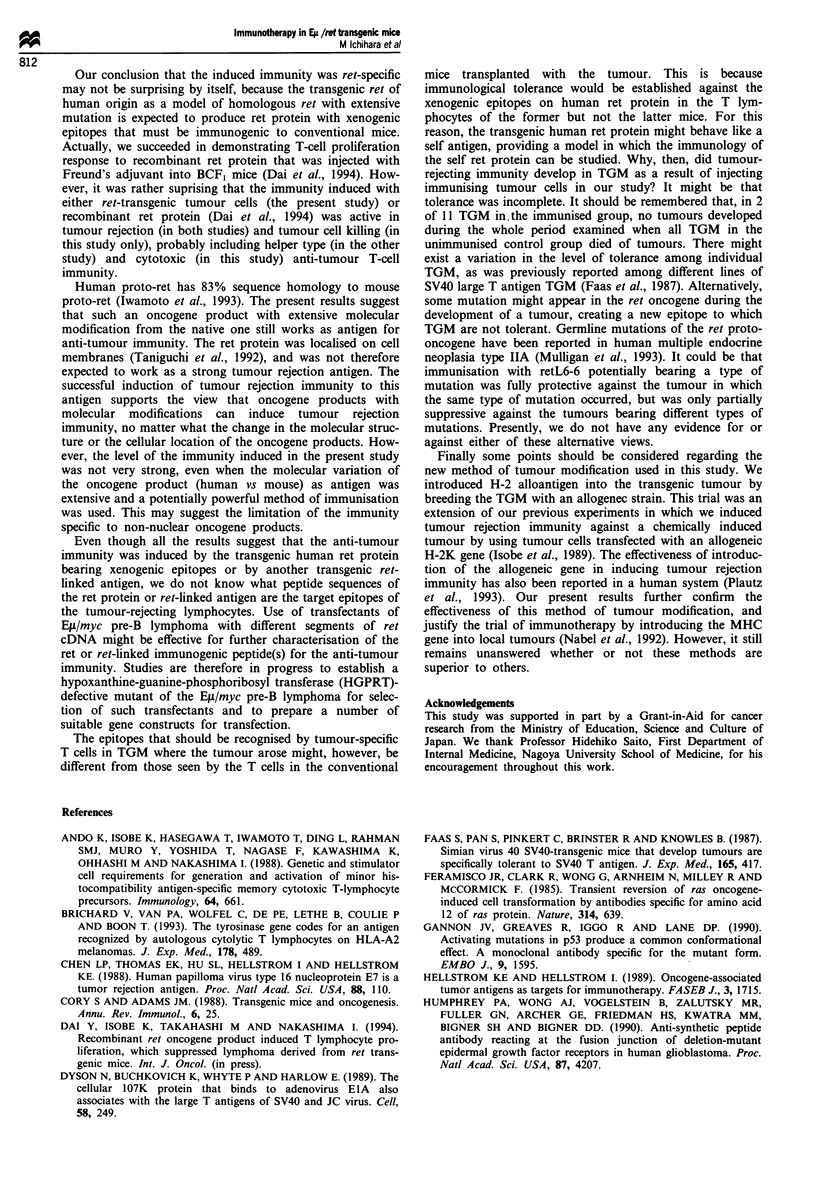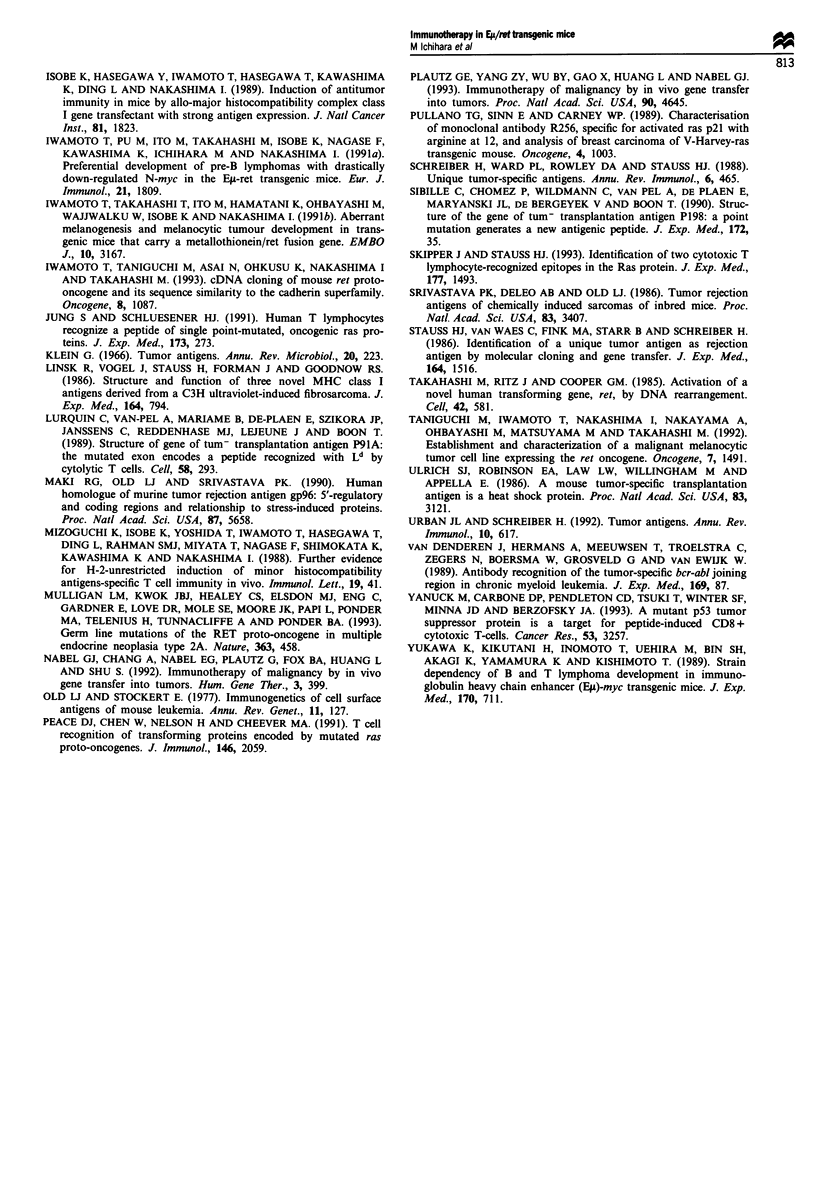# Oncogene-linked in situ immunotherapy of pre-B lymphoma arising in E mu/ret transgenic mice.

**DOI:** 10.1038/bjc.1995.156

**Published:** 1995-04

**Authors:** M. Ichihara, T. Iwamoto, K. Isobe, M. Takahashi, A. Nakayama, M. Pu, Y. Dai, A. Parashar, K. Ohkus, M. Kato

**Affiliations:** Department of Immunology, Nagoya University School of Medicine, Japan.

## Abstract

**Images:**


British Journal of Cancer (1995) 71, 808-813
© 1995 Stockton Press All rights reserved 0007-0920/95 $12.00

### Oncogene-linked *in situ* immunotherapy of pre-B lymphoma arising in Eμ/*ret* transgenic mice

M Ichihara[1,2], T Iwamoto[1], K Isobe[1], M Takahashi[3], A Nakayama[3], M Pu[1], Y Dai[1], A Parashar[1], K Ohkus[1], M Kato[1], T Hotta[2] and I Nakashima[1]

[1]*Department of Immunology,* [2]*1st Department of Internal Medicine and* [3]*Department of Pathology, Nagoya University School of Medicine, 65 Tsurumai-cho, Showa-ku, Nagoya 466, Japan.*

**Summary** We attempted to induce anti-tumour immunity for rejecting pre-B lymphoma derived from Eμ/*ret* transgenic mice (TGM). We established pre-B-lymphoma cell lines of C57BL/6 × Balb/c background ($H-2^{b/d}$) into which $H-2^k$ alloantigen and C3H background were introduced (retL1-6 and retL6-6), and we inoculated $BCF_1$ mice with these immunising tumour cells. After these tumours were rejected by alloantigen ($H-2^k$/C3H background)-specific effector cells, the mice were challenged with the pre-B-lymphoma cell line derived from the original Eμ/*ret* TGM (ret0-2). All non-immunised control mice died within 80 days, whereas half the immunised mice survived for over 300 days. The immunity was also effective against primary pre-B-lymphoma cells from Eμ/*ret* TGM and the *ret*-driven melanoma cell line (MEL-ret), but not against the pre-B-lymphoma cell line from Eμ/*myc* TGM. This immunity was at least in part mediated by cell-mediated cytotoxicity that was specific to the *ret* oncogene product or *ret*-regulated antigen. Next we immunised Eμ/*ret* TGM by inoculating them with retL6-6 cells once every 2 weeks beginning at the age of 1 month. Interestingly, this immunisation enabled the TGM to survive longer than the non-immunised control group ($P<0.05$). Moreover, 2 of 11 transgenic mice receiving such immunisation were free from both macroscopic and microscopic tumours at the time when all of the 12 non-immunised control TGM had died from their tumour. This provides a new model for oncogene-linked immunotherapy research.

**Keywords:** oncogene *ret*; transgenic mouse; pre-B lymphoma; immunotherapy

Different types of tumour antigens have been found on malignant tumours in humans and laboratory animals (Klein, 1966; Old and Stockert, 1977; Schreiber *et al.*, 1988; Urban and Schreiber, 1992). Many of them are simply recognised by antibodies and helper T cells, and only some of them act in rejecting tumours. The former type of antigens are useful for the diagnosis of tumours and for targeting tumours with toxic reagents, but only the latter are active in the immunological surveillance of tumours and can be best used for therapeutic and prophylactic purposes. Well-known tumour rejection antigens are nuclear antigens of tumour viruses such as SV40 T antigen (Klein, 1966), adenovirus E1A antigen (Dyson *et al.*, 1992) and papillomavirus type 16 nucleoprotein (Chen *et al.*, 1988), proteolysed fragments (peptides) of which can associate with class I major histocompatibility complex (MHC) antigens to be recognised by cytotoxic T lymphocytes (CTL). So-called unique antigens of chemically induced tumours and ultraviolet-induced tumours (Schreiber *et al.*, 1988) also play a role in tumour rejection. Some of them have been characterised as the 85 kDa (Ulrich *et al.*, 1986) and 96 kDa (Srivastava *et al.*, 1986) stress-induced proteins or unique class I MHC antigens (Linsk *et al.*, 1986; Stauss *et al.*, 1986) or P91A (Lurquin *et al.*, 1989) and P198 (Sibille *et al.*, 1990) with mutations of a single nucleotide. Little is known, however, about the potential tumour rejection antigens occurring on spontaneously arising tumours in humans and laboratory animals, although the antigen recognised by autologous CTL on human melanomas has been recently shown to be encoded by the tyrosinase gene (Brichard *et al.*, 1993), and a human homologue of the murine rejection antigen gp96 has been reported (Maki *et al.*, 1990).

Recent progress in oncology has revealed the multistep actions of oncogenes in oncogenesis (Cory and Adams, 1988). The products of these oncogenes may be antigenic because of the occurrence of point mutations, deletion mutations or chromosomal translocation (Hellstrom and Hellstrom, 1989; Urban and Schreiber, 1992). There are many reported examples of tumour-specific antigens closely linked to oncogene proteins, such as mutated ras p21 (Feramisco *et al.*, 1985; Pullano *et al.*, 1989; Jung and Schluesener, 1991; Peace *et al.*, 1991), the bcl-abl fusion protein (Van Denderen *et al.*, 1989), mutated p53 (Gannon *et al.*, 1990) and the deletion mutants of epidermal growth factor receptor (Humphrey *et al.*, 1990). These tumour antigens are, in most cases, recognised by antibodies and helper T cells for antibody production. Exceptions are mutated p53, which is a nuclear suppressor gene product (Yanuck *et al.*, 1993), and mutated ras proteins produced by recombinant vaccina viruses (Skipper and Stauss, 1993), which are recognised by CTL. So far, it is not known whether non-nuclear oncogene proteins in native tumour cells will act as tumour rejection antigens and whether only restricted mutational changes of the proteins can induce tumour rejection immunity.

We recently established Eμ/*ret* (Iwamoto *et al.*, 1991*a*) and MT/*ret* (Iwamoto *et al.*, 1991*b*) transgenic mice (TGM), in which pre-B lymphoma and melanocytic tumours developed respectively. The *ret* oncogene, introduced into the TGM, is a fusion gene of the protein tyrosine kinase proto-*ret*, whose sequence coding the receptor domain is replaced with another gene named *rfp* (Takahashi *et al.*, 1985), and the Ret protein is expressed in association with cell membrane (Taniguchi *et al.*, 1992). In order to determine the answers to the two questions posed above, we tried to induce rejection immunity against these tumours. A new method was used to modify the transgenic tumour for immunisation; modified tumour cell lines were established from the tumour arising in the transgenic mice into which alloantigens were introduced by crossing. The results show that the *ret* oncogene is active in inducing anti-tumour immunity which is effective in rejecting both transgenic tumours transplanted into otherwise genetically compatible hosts and tumours arising in transgenic mice *in situ*.

Correspondence: I Nakashima
Received 13 July 1994; revised 14 November 1994; accepted 15 November 1994

### Materials and methods

### *Mice*

C57BL/6 × Balb/c $F_1$ ($BCF_1$) (H-$2^{b/d}$) and Balb/c females were bred in the Institute for Animal Research, Nagoya University School of Medicine, or purchased from Shizuoka Agricultural Center, Hamamatsu, Shizuoka. The Eμ/*ret* TGM were previously established from $BCF_1$ mice (Iwamoto *et al.*, 1991*a*). Eμ/*ret* TGM were bred with Balb/c, and the progeny containing the transgene were selected as previously described (Iwamoto *et al.*, 1991*a*).

### *Cells*

ret0-2 was established from lymphoma developed in an Eμ/*ret* TGM. MEL-ret (Taniguchi *et al.*, 1992), a melanoma cell line, was derived from a metallothionein/*ret* TGM (Iwamoto *et al.*, 1991*b*). An Eμ/*myc* pre-B-lymphoma cell line of C57BL/6 background (Yukawa *et al.*, 1989) was kindly donated by Dr Yukawa (Institute for Molecular and Cellular Biology, Osaka University). These cell lines, which are listed in Table I together with their H-2 haplotypes, were used as challenging tumours to the previously immunised $BCF_1$ mice.

### *Establishment of immunising tumour cell lines*

In our previous study, we induced a strong anti-tumour immunity to original tumour cells by immunisation with the tumour cells xenogenised by introducing allogeneic MHC Class I gene (Isobe *et al.*, 1989). Instead of transfecting with the allogeneic MHC class I gene, we bred the Eμ/*ret* TGM with C3H/HeJ mice expressing H-$2^k$ antigen to obtain lymphoma cell lines possessing the same character as ret0-2 except for the H-$2^k$/C3H background expression. We established two lymphoma cell lines (H-$2^{d/k}$), retL1-6 and retL6-6, as immunising tumour cells (Table I).

### *Immunisation procedure*

$BCF_1$ male mice (2–3 months old) received i.p. injections of $1 \times 10^7$ immunising tumour cells twice in a month. Two weeks after the last i.p. injection, challenging tumour cells were injected i.p. into the mice and the survival time was estimated as compared with that of control mice that did not receive immunising tumour cells.

We also evaluated the effects of immunisation on tumour development in Eμ/*ret* TGM. A group of Eμ/*ret* TGM received repeated i.p. injections of $1 \times 10^7$ immunising tumour cells once every 2 weeks from the age of 1 month. Survival times from their birth were compared with those of control TGM that did not receive immunising tumour cells. Some mice were sacrificed and examined pathologically.

### *Assay for cell-mediated cytotoxicity*

Spleen cells suspended in RPMI-1640 medium containing 10% fetal calf serum were sensitised *in vitro* with tumour cells irradiated with 1500 rad at a stimulator-to-target ratio of 1:10 for 4 days. The cytotoxicity of these cells was measured by the $^{51}$Cr-release assay. Target cells were labelled with $^{51}$Cr, mixed with effector cells and incubated for 4 h at 37°C. The supernatant was collected for measurement of radioactivity by a gamma scintillation counter. The percentage of specific lysis was calculated as follows:

$$\text{Specific lysis (\%)} = (\text{experimental c.p.m.} - \text{spontaneous c.p.m.})/(\text{maximum c.p.m.} - \text{spontaneous c.p.m.}) \times 100$$

**Table I** A list of transgenic tumour cell lines used in this study

| *Cell line* | *Transgenic oncogene* | *H-2* | *Used for* |
|---|---|---|---|
| ret0-2 | *ret* | b/d | Challenge *in vivo* |
| | | | Target of *in vitro* killing |
| retL1-6 | *ret* | k/d | Immunisation |
| retL6-6 | *ret* | k/d | Immunisation |
| | | | Target of *in vitro* killing |
| MEL-ret | *ret* | b | Challenge *in vivo* |
| | | | Target of *in vitro* killing |
| Eμ/*myc* pre-B | *myc* | b | Challenge *in vivo* |
| | | | Target of *in vitro* killing |

### *Statistical analysis*

The survival rate was calculated using the Kaplan–Meier method. Statistical analysis was based on generalised Wilcoxon tests. Values were expressed as the mean ± standard deviation (s.d.).

### Results

### *Characterisation of the immunising/challenging tumour cell lines*

We first examined the characteristics of the immunising/challenging tumour cell lines. The negative cell-surface Ig expression and the selective rearrangement of the immunoglobulin heavy-chain genes indicated that the cell lines ret0-2, retL1-6 and retL6-6 had the pre-B-lymphoma phenotype. A difference in the rearranged band size of the immunoglobulin gene between retL1-6 and retL6-6 showed that these lymphoma cell lines were different clones (data not shown). As might be anticipated, these two cell lines, which expressed H-$2^k$ antigen, were completely rejected in $BCF_1$ mice after i.p. inoculation (data not shown).

### *Induction of anti-tumour immunity rejecting* ret *TGM-derived tumour cells*

Figure 1 shows the survival time of mice previously immunised with retL1-6 or retL6-6 after challenging with $2 \times 10^6$ ret0-2 cells. Both retL1-6 and retL6-6 cell lines induced anti-tumour immunity, rejecting ret0-2 cells. One-half to two-thirds of the mice survived more than 300 days after challenge. On the other hand, all the mice that had not been immunised died within 100 days. This difference was statistically significant ($P<0.01$). These data demonstrated that two different lymphoma cell lines (retL1-6, retL6-6) independently induced anti-tumour immunity to the other lymphoma cell line (ret0-2). When we inoculated $1 \times 10^7$ of retL0-2 cells into the immunised mice, however, there was no statistically significant difference in the survival time between immunised and non-immunised mice, although the survival time of immunised mice was slightly longer than that of the

**Figure 1** Induction of anti-tumour immunity rejecting TGM-derived tumour cells in $BCF_1$ mice. RetL1-6 and retL6-6 cells at ($1 \times 10^7$) were injected intraperitoneally (i.p.) into $BCF_1$ mice twice in a month. Two weeks after the last injection, ret0-2 cells were challenged at $2 \times 10^6$ i.p. into these mice and into non-immunised control mice. Survival rate was recorded in days post tumour challenge.

non-immunised mice (data not shown). This result showed that the immunity induced was not very strong.

Next, we evaluated the specificity of the anti-tumour immunity induced by retL6-6 (Figure 2). Even when challenged by primary lymphoma cells from Eμ/*ret* TGM, immunised mice survived longer ($P<0.05$) than non-immunised mice. Interestingly, the anti-tumour immunity induced by immunisation with Eμ/*ret* B-lymphoma cells was also directed against MEL-ret melanoma cells, while no significant effect was observed on Eμ/*myc* TGM-derived pre-B-lymphoma cells. These data suggested that the induced anti-tumour immunity was specific to the *ret* oncogene product or its closely related antigen.

A further study was conducted to determine whether cell-mediated cytotoxicity was induced by the immunising protocol. After inoculation of retL6-6 cells and ret0-2 cells into mice and *in vitro* secondary sensitisation of spleen cells from these mice with ret0-2 cells, the effector activity developed to kill ret0-2 cells in addition to retL6-6 cells. The effector cells also killed MEL-ret cells, but not Eμ/*myc* TGM-derived pre-B-lymphoma cells (Table II). These results confirmed the specificity of the induced immunity against *ret* TGM-derived tumours.

**Figure 2** Specificity of the induced anti-tumour immunity. After immunisation with retL6-6 according to the same procedure described in Figure 1, primary tumour cells from Eμ/*ret* TGM (H-$2^b$) at $5 \times 10^6$ (**a**), MEL-ret, melanocyte tumour cell line at $4 \times 10^6$ (**b**) and Eμ/*myc* TGM-derived pre-B-lymphoma cells at $2 \times 10^4$ (**c**) were injected i.p. into the immunised and unimmunised control $BCF_1$ mice. Survival rate was recorded in days post tumour challenge.

**Table II** Specificity of the *in vitro* cytotoxicity induced by the immunisation with retL-6-6

| | $^{51}$CR release (%) against | | | |
|---|---|---|---|---|
| *E/T ratio* | *ret0-2* | *MEL*-ret | *Eμ*/myc *pre-B* | *retL6-6* |
| 50 | 28.2 + 2.0 | 25.6 + 2.2 | 0 + 0 | 52.3 + 2.5 |
| 10 | 16.5 + 1.9 | 15.8 + 1.7 | 0 + 0 | 12.7 + 1.6 |
| 1 | 1.9 + 2.7 | 4.1 + 0.7 | 0 + 0 | 0.1 + 0 |

RetL6-6 immunised mice were inoculated twice with $2 \times 10^6$ ret0-2 cells. The spleen cells obtained from these mice 1 week after the last injection of ret0-2 were sensitised *in vitro* with ret0-2 and assayed for the lytic activity against the target cells.

*Suppression of tumour development in Eμ-*ret *TGM by immunisation*

As the final goal of this immunisation, we tested whether this immunisation procedure could suppress tumour development in Eμ/*ret* TGM *in situ*. Figure 3 shows the survival time of the TGM which had or had not been immunised with retL6-6 cells. This immunisation enabled the TGM to survive longer than the non-immunised control group ($P<0.05$).

Autopsies were performed on all mice tested. In the non-immunised control group, all 12 TGM died from progression of lymphoma by day 174 after birth. In the immunised group, however, 8 of 11 (73%) TGM survived until day 174, three of which showed no evidence of lymphoma development on superficial examination. Two of these TGM were sacrificed, and another died of severe general emaciation, for reasons unknown. Pathological examination revealed that one of them had a lymphomatous mass in the abdomen, but the other two were pathologically free of lymphoma development (Figure 4a–c). This contrasted with extensive proliferation of lymphoma cells observed in the lymph nodes (Figure 4d and e) and bone marrow (Figure 4f) of all mice in the control group. Transmission of the *ret* gene into those lymphoma-free mice was confirmed by repeated testing from tail DNA.

### Discussion

This study shows that pre-B-lymphoma cells arising in the Eμ/*ret* transgenic mouse carry tumour antigens which induce anti-tumour immunity for both *in vivo* tumour rejection and *in vitro* tumour cell killing. This immunity was not as strong as that produced by virally or chemically induced tumours. However, the immunity was effective not only in prolonging the survival of mice transplanted with the tumour, but also in suppressing the primary development of the tumour in the TGM *in situ* (Figure 3). The latter finding is particularly notable because it for the first time provides evidence that cellular oncogene-induced tumours may be subject to oncogene product-linked immunological surveillance.

Analysis using four different types of *ret*-transgenic tumour cells as the target of the immunity, all of which except the transgenic *ret* were genetically compatible with the host immune system, demonstrated that the immunity was *ret*-specific or *ret*-linked. Three types of *ret* transgenic tumour

**Figure 3** Suppression of tumour development in Eμ/*ret* TGM by immunisation. Eμ/*ret* TGM received repeated i.p. injections of immunising $1 \times 10^7$ retL6-6 cells once every 2 weeks from the age of 1 month. Survival times from their birth were evaluated as compared with those of control Eμ/*ret* TGM that did not receive immunising tumour cells. Two mice from the immunising group (◆) which were free of lymphoma development on superficial observation were sacrificed and examined pathologically. Triangles in this figure indicate the times when retL6-6 cells were injected into TGM.

**Figure 4** Complete suppression of tumour development in some TGM of the immunised group. The histology of the lymph node and bone marrow from one of the two TGM sacrificed on day 174 (see Figure 3) and a control non-immunised TGM that died on that day is shown. Note that there is no evidence of lymphoma development in the normal structure of the peripheral lymph node with primary and secondary follicles (**d** and **e**) and bone marrow (**f**) from the immunised TGM, which contrasted with extensive proliferation of lymphoma cells in the peripheral lymph node (**a** and **b**) and bone marrow (**c**) from the non-immunised control TGM, destroying the normal structure. Stained with haematoxylin–eosin. **a** and **d**, × 17; **b**, **c**, **e** and **f**, × 170.

cells from different transgenic individuals, the Eμ/*ret* pre-B-lymphoma cell line (H-$2^{b/d}$), Eμ/*ret* primary pre-B lymphoma (H-$2^b$) and the MEL-ret melanoma cell line (H-$2^b$), were susceptible to the anti-tumour immunity. This suggested that the induced immunity was directed against the *ret*-linked antigen or the ret protein itself, which should be the only potential candidate immunogenic element shared by the three tumour cells as all other genetically determined antigens (H-2 and minor) of these tumours of B6 × Balb/c origin should not be antigenic for the host $F_1$ mice of B6 and Balb/c strains. In agreement with this conclusion, the induced immunity was not directed to Eμ/*myc* pre-B lymphoma (H-$2^b$), which differs only at the transgenic oncogene from Eμ/*ret* primary pre-B lymphoma (H-$2^b$), except for some background genes (B6 for Eμ/*myc* and B6 × Balb/c for Eμ/*ret*) whose products should not be antigenic in $F_1$ mice according to the accepted transplantation immunology rule.

The *ret*-linked anti-tumour immunity was induced by priming with alloantigen (H-$2^{k/d}$) bearing *ret*-transgenic cells (retL1-6 or retL6-6). However, the induced immunity was not restricted by the H-2 of the cells for priming, protecting against the challenge of both H-$2^{b/d}$ and H-$2^b$ *ret*-transgenic tumours. This finding corresponded to our previous result that priming for the secondary CTL responses to non-H-2 cellular antigens *in vivo* (Mizoguchi *et al.*, 1988) and *in vitro* (Ando *et al.*, 1988) is not restricted by the H-2 of the immunising cells for priming, suggesting effective processing of the tumour antigen by host cells for priming.

Our conclusion that the induced immunity was *ret*-specific may not be surprising by itself, because the transgenic *ret* of human origin as a model of homologous *ret* with extensive mutation is expected to produce ret protein with xenogenic epitopes that must be immunogenic to conventional mice. Actually, we succeeded in demonstrating T-cell proliferation response to recombinant ret protein that was injected with Freund's adjuvant into $BCF_1$ mice (Dai *et al.*, 1994). However, it was rather suprising that the immunity induced with either *ret*-transgenic tumour cells (the present study) or recombinant ret protein (Dai *et al.*, 1994) was active in tumour rejection (in both studies) and tumour cell killing (in this study only), probably including helper type (in the other study) and cytotoxic (in this study) anti-tumour T-cell immunity.

Human proto-ret has 83% sequence homology to mouse proto-ret (Iwamoto *et al.*, 1993). The present results suggest that such an oncogene product with extensive molecular modification from the native one still works as antigen for anti-tumour immunity. The ret protein was localised on cell membranes (Taniguchi *et al.*, 1992), and was not therefore expected to work as a strong tumour rejection antigen. The successful induction of tumour rejection immunity to this antigen supports the view that oncogene products with molecular modifications can induce tumour rejection immunity, no matter what the change in the molecular structure or the cellular location of the oncogene products. However, the level of the immunity induced in the present study was not very strong, even when the molecular variation of the oncogene product (human *vs* mouse) as antigen was extensive and a potentially powerful method of immunisation was used. This may suggest the limitation of the immunity specific to non-nuclear oncogene products.

Even though all the results suggest that the anti-tumour immunity was induced by the transgenic human ret protein bearing xenogenic epitopes or by another transgenic *ret*-linked antigen, we do not know what peptide sequences of the ret protein or *ret*-linked antigen are the target epitopes of the tumour-rejecting lymphocytes. Use of transfectants of $E\mu$/*myc* pre-B lymphoma with different segments of *ret* cDNA might be effective for further characterisation of the ret or *ret*-linked immunogenic peptide(s) for the anti-tumour immunity. Studies are therefore in progress to establish a hypoxanthine-guanine-phosphoribosyl transferase (HGPRT)-defective mutant of the $E\mu$/*myc* pre-B lymphoma for selection of such transfectants and to prepare a number of suitable gene constructs for transfection.

The epitopes that should be recognised by tumour-specific T cells in TGM where the tumour arose might, however, be different from those seen by the T cells in the conventional mice transplanted with the tumour. This is because immunological tolerance would be established against the xenogenic epitopes on human ret protein in the T lymphocytes of the former but not the latter mice. For this reason, the transgenic human ret protein might behave like a self antigen, providing a model in which the immunology of the self ret protein can be studied. Why, then, did tumour-rejecting immunity develop in TGM as a result of injecting immunising tumour cells in our study? It might be that tolerance was incomplete. It should be remembered that, in 2 of 11 TGM in the immunised group, no tumours developed during the whole period examined when all TGM in the unimmunised control group died of tumours. There might exist a variation in the level of tolerance among individual TGM, as was previously reported among different lines of SV40 large T antigen TGM (Faas *et al.*, 1987). Alternatively, some mutation might appear in the *ret* oncogene during the development of a tumour, creating a new epitope to which TGM are not tolerant. Germline mutations of the *ret* proto-oncogene have been reported in human multiple endocrine neoplasia type IIA (Mulligan *et al.*, 1993). It could be that immunisation with retL6-6 potentially bearing a type of mutation was fully protective against the tumour in which the same type of mutation occurred, but was only partially suppressive against the tumours bearing different types of mutations. Presently, we do not have any evidence for or against either of these alternative views.

Finally some points should be considered regarding the new method of tumour modification used in this study. We introduced H-2 alloantigen into the transgenic tumour by breeding the TGM with an allogenec strain. This trial was an extension of our previous experiments in which we induced tumour rejection immunity against a chemically induced tumour by using tumour cells transfected with an allogeneic H-2K gene (Isobe *et al.*, 1989). The effectiveness of introduction of the allogeneic gene in inducing tumour rejection immunity has also been reported in a human system (Plautz *et al.*, 1993). Our present results further confirm the effectiveness of this method of tumour modification, and justify the trial of immunotherapy by introducing the MHC gene into local tumours (Nabel *et al.*, 1992). However, it still remains unanswered whether or not these methods are superior to others.

**Acknowledgements**
This study was supported in part by a Grant-in-Aid for cancer research from the Ministry of Education, Science and Culture of Japan. We thank Professor Hidehiko Saito, First Department of Internal Medicine, Nagoya University School of Medicine, for his encouragement throughout this work.

**References**

ANDO K, ISOBE K, HASEGAWA T, IWAMOTO T, DING L, RAHMAN SMJ, MURO Y, YOSHIDA T, NAGASE F, KAWASHIMA K, OHHASHI M AND NAKASHIMA I. (1988). Genetic and stimulator cell requirements for generation and activation of minor histocompatibility antigen-specific memory cytotoxic T-lymphocyte precursors. *Immunology*, **64**, 661.

BRICHARD V, VAN PA, WOLFEL C, DE PE, LETHE B, COULIE P AND BOON T. (1993). The tyrosinase gene codes for an antigen recognized by autologous cytolytic T lymphocytes on HLA-A2 melanomas. *J. Exp. Med.*, **178**, 489.

CHEN LP, THOMAS EK, HU SL, HELLSTROM I AND HELLSTROM KE. (1988). Human papilloma virus type 16 nucleoprotein E7 is a tumor rejection antigen. *Proc. Natl Acad. Sci. USA*, **88**, 110.

CORY S AND ADAMS JM. (1988). Transgenic mice and oncogenesis. *Annu. Rev. Immunol.*, **6**, 25.

DAI Y, ISOBE K, TAKAHASHI M AND NAKASHIMA I. (1994). Recombinant *ret* oncogene product induced T lymphocyte proliferation, which suppressed lymphoma derived from *ret* transgenic mice. *Int. J. Oncol.* (in press).

DYSON N, BUCHKOVICH K, WHYTE P AND HARLOW E. (1989). The cellular 107K protein that binds to adenovirus E1A also associates with the large T antigens of SV40 and JC virus. *Cell*, **58**, 249.

FAAS S, PAN S, PINKERT C, BRINSTER R AND KNOWLES B. (1987). Simian virus 40 SV40-transgenic mice that develop tumours are specifically tolerant to SV40 T antigen. *J. Exp. Med.*, **165**, 417.

FERAMISCO JR, CLARK R, WONG G, ARNHEIM N, MILLEY R AND MCCORMICK F. (1985). Transient reversion of *ras* oncogene-induced cell transformation by antibodies specific for amino acid 12 of *ras* protein. *Nature*, **314**, 639.

GANNON JV, GREAVES R, IGGO R AND LANE DP. (1990). Activating mutations in p53 produce a common conformational effect. A monoclonal antibody specific for the mutant form. *EMBO J.*, **9**, 1595.

HELLSTROM KE AND HELLSTROM I. (1989). Oncogene-associated tumor antigens as targets for immunotherapy. *FASEB J.*, **3**, 1715.

HUMPHREY PA, WONG AJ, VOGELSTEIN B, ZALUTSKY MR, FULLER GN, ARCHER GE, FRIEDMAN HS, KWATRA MM, BIGNER SH AND BIGNER DD. (1990). Anti-synthetic peptide antibody reacting at the fusion junction of deletion-mutant epidermal growth factor receptors in human glioblastoma. *Proc. Natl Acad. Sci. USA*, **87**, 4207.

ISOBE K, HASEGAWA Y, IWAMOTO T, HASEGAWA T, KAWASHIMA K, DING L AND NAKASHIMA I. (1989). Induction of antitumor immunity in mice by allo-major histocompatibility complex class I gene transfectant with strong antigen expression. *J. Natl Cancer Inst.*, **81**, 1823.

IWAMOTO T, PU M, ITO M, TAKAHASHI M, ISOBE K, NAGASE F, KAWASHIMA K, ICHIHARA M AND NAKASHIMA I. (1991*a*). Preferential development of pre-B lymphomas with drastically down-regulated N-*myc* in the Eμ-ret transgenic mice. *Eur. J. Immunol.*, **21**, 1809.

IWAMOTO T, TAKAHASHI T, ITO M, HAMATANI K, OHBAYASHI M, WAJJWALKU W, ISOBE K AND NAKASHIMA I. (1991*b*). Aberrant melanogenesis and melanocytic tumour development in transgenic mice that carry a metallothionein/ret fusion gene. *EMBO J.*, **10**, 3167.

IWAMOTO T, TANIGUCHI M, ASAI N, OHKUSU K, NAKASHIMA I AND TAKAHASHI M. (1993). cDNA cloning of mouse *ret* proto-oncogene and its sequence similarity to the cadherin superfamily. *Oncogene*, **8**, 1087.

JUNG S AND SCHLUESENER HJ. (1991). Human T lymphocytes recognize a peptide of single point-mutated, oncogenic ras proteins. *J. Exp. Med.*, **173**, 273.

KLEIN G. (1966). Tumor antigens. *Annu. Rev. Microbiol.*, **20**, 223.

LINSK R, VOGEL J, STAUSS H, FORMAN J AND GOODNOW RS. (1986). Structure and function of three novel MHC class I antigens derived from a C3H ultraviolet-induced fibrosarcoma. *J. Exp. Med.*, **164**, 794.

LURQUIN C, VAN-PEL A, MARIAME B, DE-PLAEN E, SZIKORA JP, JANSSENS C, REDDENHASE MJ, LEJEUNE J AND BOON T. (1989). Structure of gene of $tum^-$ transplantation antigen P91A: the mutated exon encodes a peptide recognized with $L^d$ by cytolytic T cells. *Cell*, **58**, 293.

MAKI RG, OLD LJ AND SRIVASTAVA PK. (1990). Human homologue of murine tumor rejection antigen gp96: 5′-regulatory and coding regions and relationship to stress-induced proteins. *Proc. Natl Acad. Sci. USA*, **87**, 5658.

MIZOGUCHI K, ISOBE K, YOSHIDA T, IWAMOTO T, HASEGAWA T, DING L, RAHMAN SMJ, MIYATA T, NAGASE F, SHIMOKATA K, KAWASHIMA K AND NAKASHIMA I. (1988). Further evidence for H-2-unrestricted induction of minor histocompatibility antigens-specific T cell immunity in vivo. *Immunol. Lett.*, **19**, 41.

MULLIGAN LM, KWOK JBJ, HEALEY CS, ELSDON MJ, ENG C, GARDNER E, LOVE DR, MOLE SE, MOORE JK, PAPI L, PONDER MA, TELENIUS H, TUNNACLIFFE A AND PONDER BA. (1993). Germ line mutations of the RET proto-oncogene in multiple endocrine neoplasia type 2A. *Nature*, **363**, 458.

NABEL GJ, CHANG A, NABEL EG, PLAUTZ G, FOX BA, HUANG L AND SHU S. (1992). Immunotherapy of malignancy by in vivo gene transfer into tumors. *Hum. Gene Ther.*, **3**, 399.

OLD LJ AND STOCKERT E. (1977). Immunogenetics of cell surface antigens of mouse leukemia. *Annu. Rev. Genet.*, **11**, 127.

PEACE DJ, CHEN W, NELSON H AND CHEEVER MA. (1991). T cell recognition of transforming proteins encoded by mutated *ras* proto-oncogenes. *J. Immunol.*, **146**, 2059.

PLAUTZ GE, YANG ZY, WU BY, GAO X, HUANG L AND NABEL GJ. (1993). Immunotherapy of malignancy by in vivo gene transfer into tumors. *Proc. Natl Acad. Sci. USA*, **90**, 4645.

PULLANO TG, SINN E AND CARNEY WP. (1989). Characterisation of monoclonal antibody R256, specific for activated ras p21 with arginine at 12, and analysis of breast carcinoma of V-Harvey-ras transgenic mouse. *Oncogene*, **4**, 1003.

SCHREIBER H, WARD PL, ROWLEY DA AND STAUSS HJ. (1988). Unique tumor-specific antigens. *Annu. Rev. Immunol.*, **6**, 465.

SIBILLE C, CHOMEZ P, WILDMANN C, VAN PEL A, DE PLAEN E, MARYANSKI JL, DE BERGEYEK V AND BOON T. (1990). Structure of the gene of $tum^-$ transplantation antigen P198: a point mutation generates a new antigenic peptide. *J. Exp. Med.*, **172**, 35.

SKIPPER J AND STAUSS HJ. (1993). Identification of two cytotoxic T lymphocyte-recognized epitopes in the Ras protein. *J. Exp. Med.*, **177**, 1493.

SRIVASTAVA PK, DELEO AB AND OLD LJ. (1986). Tumor rejection antigens of chemically induced sarcomas of inbred mice. *Proc. Natl. Acad. Sci. USA*, **83**, 3407.

STAUSS HJ, VAN WAES C, FINK MA, STARR B AND SCHREIBER H. (1986). Identification of a unique tumor antigen as rejection antigen by molecular cloning and gene transfer. *J. Exp. Med.*, **164**, 1516.

TAKAHASHI M, RITZ J AND COOPER GM. (1985). Activation of a novel human transforming gene, *ret*, by DNA rearrangement. *Cell*, **42**, 581.

TANIGUCHI M, IWAMOTO T, NAKASHIMA I, NAKAYAMA A, OHBAYASHI M, MATSUYAMA M AND TAKAHASHI M. (1992). Establishment and characterization of a malignant melanocytic tumor cell line expressing the *ret* oncogene. *Oncogene*, **7**, 1491.

ULRICH SJ, ROBINSON EA, LAW LW, WILLINGHAM M AND APPELLA E. (1986). A mouse tumor-specific transplantation antigen is a heat shock protein. *Proc. Natl Acad. Sci. USA*, **83**, 3121.

URBAN JL AND SCHREIBER H. (1992). Tumor antigens. *Annu. Rev. Immunol.*, **10**, 617.

VAN DENDEREN J, HERMANS A, MEEUWSEN T, TROELSTRA C, ZEGERS N, BOERSMA W, GROSVELD G AND VAN EWIJK W. (1989). Antibody recognition of the tumor-specific *bcr-abl* joining region in chronic myeloid leukemia. *J. Exp. Med.*, **169**, 87.

YANUCK M, CARBONE DP, PENDLETON CD, TSUKI T, WINTER SF, MINNA JD AND BERZOFSKY JA. (1993). A mutant p53 tumor suppressor protein is a target for peptide-induced CD8+ cytotoxic T-cells. *Cancer Res.*, **53**, 3257.

YUKAWA K, KIKUTANI H, INOMOTO T, UEHIRA M, BIN SH, AKAGI K, YAMAMURA K AND KISHIMOTO T. (1989). Strain dependency of B and T lymphoma development in immunoglobulin heavy chain enhancer (Eμ)-*myc* transgenic mice. *J. Exp. Med.*, **170**, 711.